# Psychometric Properties of the PHQ-9 and CESD-R Depression Measures with Older Adults

**DOI:** 10.1093/geroni/igaa057.3258

**Published:** 2020-12-16

**Authors:** Emma Katz, Rachael Spalding, Barry Edelstein

**Affiliations:** West Virginia University, Morgantown, West Virginia, United States

## Abstract

Our understanding of older adult depression has been impeded by the paucity of assessment instruments with validity evidence for older adults. Therefore, measures of depression that were initially developed for use with younger adults are commonly used with older adults as well, such as the Center for Epidemiological Studies Depression Scale-Revised (CESD-R; Eaton et al., 2004) and Patient Health Questionnaire-9 (PHQ-9; Kroenke, Spitzer, & Williams, 2001). The CESD-R (Jiang et al., 2019; Van Dam & Earleywine, 2011) and PHQ-9 (Indu et al., 2018; Levis, Benedetti, & Thombs, 2019) have strong psychometric support for their use with young adults, and are two frequently used depression measures. In light of age-related differences in the experience and presentation of depression (e.g., Balsamo, et al., 2015; Fiske, Wetherell, & Gatz, 2009; Hybels, Laderman, & Blazer, 2012; Wuthrich, Johnco & Wetherell, 2015), the present study examined the psychometric properties of these instruments with older adults. Two-hundred-and-seventy-seven older adults (ages 65 and older) completed an online survey including the PHQ-9, CESD-R, and instruments measuring several other constructs with anticipated relations to depression (anxiety, general depression, positive and negative affect, self-esteem, personality traits, and satisfaction with life). The relation between the two depression scales and measures of the other constructs were examined. Both the PHQ-9 and CESD-R evidenced good internal consistency reliability (a = .82 and .83, respectively) and strong correlations in anticipated directions with many of the related constructs. These results support the use of the CESD-R and PHQ-9 with community-dwelling older adults.

